# Triple-Therapy of Peritoneal Metastasis—Partial-Dehydration under Hyperthermic Condition Combined with Chemotherapy: The First Preliminary In-Vitro Results

**DOI:** 10.3390/ph16050763

**Published:** 2023-05-18

**Authors:** Carolina Khosrawipour, Agata Diakun, Shiri Li, Hien Lau, Joanna Kulas, Veria Khosrawipour, Wojciech Kielan, Agata Mikolajczyk-Martinez

**Affiliations:** 1 Faculty of Medicine, Wroclaw Medical University, 50-367 Wroclaw, Poland; 2 2nd Department of General Surgery and Surgical Oncology, Wroclaw Medical University, 50-367 Wroclaw, Poland; 3 Division of Colon and Rectal Surgery, Department of Surgery, New York Presbyterian Hospital-Weill Cornell College of Medicine, New York, NY 10065, USA; 4 Department of Surgery, University of California Irvine (UCI)—Medical Center, Irvine, CA 92868, USA; 5 Faculty of Veterinary Medicine, Wroclaw University of Environmental and Life Sciences, 50-375 Wroclaw, Poland; 6 Department of Surgery, Petrus-Hospital Wuppertal, 42283 Wuppertal, Germany; 7 Department of Biochemistry and Molecular Biology, Faculty of Veterinary Medicine, Wroclaw University of Environmental and Life Sciences, 50-375 Wroclaw, Poland

**Keywords:** peritoneal metastasis, colorectal cancer, intraperitoneal chemotherapy, dehydration, hyperthermia, oxaliplatin, doxorubicin

## Abstract

A newly introduced combination of intraperitoneal dehydration and hyperthermia has recently been shown to be feasible and cytotoxic for colon cancer cells in vivo. For the first time, our study now aims to evaluate dehydration under hyperthermic conditions combined with chemotherapy for potential use in the clinical setting. In this study, in vitro colon cancer cells (HT-29) were subjected to single or several cycles of partial dehydration under hyperthermic conditions (45 °C), followed by chemotherapy (triple exposure) with oxaliplatin or doxorubicin in various configurations. The viability, cytotoxicity, and proliferation of cells after the proposed protocols were assessed. Intracellular doxorubicin uptake was measured via flow cytometry. After one cycle of triple exposure, the viability of HT-29 cells was significantly reduced versus the untreated control (65.11 ± 5%, *p* < 0.0001) and versus only chemotherapy (61.2 ± 7%, *p* < 0.0001). An increased chemotherapeutic inflow into the cells after triple exposure was detected (53.4 ± 11%) when compared to cells treated with chemotherapy alone (34.23 ± 10%) (*p* < 0.001). Partial dehydration in a hyperthermic condition combined with chemotherapy increases the overall cytotoxicity of colon cancer cells significantly compared to chemotherapy alone. This could possibly be related to enhanced intracellular uptake of chemotherapeutic agents after partial dehydration. Further studies are required for the further evaluation of this new concept.

## 1. Introduction

**Figure 1:**
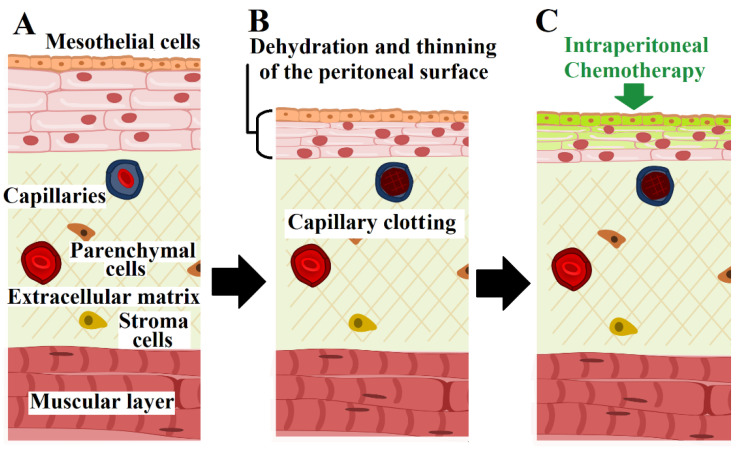
Model of laparoscopically induced peritoneal surface-dehydration under hyperthermic conditions followed by intraperitoneal chemotherapy, based on Diakun A. et al. (2022). From A to B: Model of physiological peritoneal tissue in (**A**) followed by laparoscopically induced partial dehydration of the surface under hyperthermic conditions (**B**). The dehydration process combined with hyperthermia induces partial clotting of the most superficial capillaries and a shrinking/thinning of the peritoneal surface (**B**). After this part of the procedure, intraperitoneal chemotherapy can be applied to target peritoneal metastasis (**C**).

The management of peritoneal metastasis (PM) in patients is a challenging problem in surgical oncology. Intraperitoneal cancer cell dissemination is usually a result of metastasis from the gastrointestinal tract and gynecological cancers and rarely develops as a primary neoplasm such as mesothelioma or pseudo-lymphoma [1,2]. The main mode of cancer cell dissemination is believed to be the spread of single, undetached cancer cells that passively move along the peritoneal surfaces. Some of these floating cancer cells adhere to the mesothelial surfaces at some point, and local cancer growth and invasion begin. Although other explanations have been discussed, this is currently the most accepted model of intraperitoneal cancer cell dissemination. Patients diagnosed with PM have a poor prognosis, and the median index patient does not survive the first years after diagnosis due to the rapid progression of cancer and its complications [3,4]. One of the major obstacles in the treatment of PM is the ineffectiveness of systemic chemotherapy due to the limited accumulation of the chemotherapeutic agent in the peritoneal tissue and thus the inability to achieve effective therapeutic drug concentrations [5,6]. Therefore, in the last decades, attempts have been made to expose PM directly to the chemotherapeutic agent in the form of intraperitoneal chemo-lavage. The best-known approach is currently the combination of cytoreduction surgery (CRS) and hyperthermic intraperitoneal chemotherapy (HIPEC). The effect of hyperthermic intraperitoneal chemotherapy enhanced the overall performance of CRS [7,8]. Although this therapeutic option has shown an improvement in the outcome for some PM patients, this potential curative option is not feasible for most PM patients. Often, patients with extensive PM do not qualify for the procedure, especially those with extensive peritoneal metastasis of the small intestine. If the CC-0 stage (which means full macroscopical removal of all cancer nodules) cannot be reached, the remaining cancer will spread easily throughout the peritoneal cavity again. Furthermore, a relevant number of peri- and post-operative challenges and risks limit the number of patients that benefit from the CRS with HIPEC [9,10]. Therefore, for the leftover group of patients, other strategies were conceived. One such new approach was the introduction of pressurized intraperitoneal aerosol chemotherapy (PIPAC) [11,12]. PIPAC is easy to apply, and it can be used for patients that were previously excluded from surgery due to extensive peritoneal metastasis. This method uses a laparoscopic procedure to introduce an aerosolized chemotherapeutic agent into the abdominal cavity [13,14]. Since this procedure is far less invasive, it gained popularity in the palliative setting of PM patients, and it is further evaluated for a potential neoadjuvant approach [15,16]. There were also several attempts to modify and use aerosolized chemotherapy in other surface malignancies [17,18,19] and apply new physical principles and different therapeutic agents in the treatment of PM [20,21,22,23]. A variety of improvements regarding the application and injection system have been proposed, and new treatment elements have been added. New spraying devices had been proposed by different research groups worldwide that were less costly and had new features such as rotating heads. In addition, new types of drug combinations have been discussed and introduced. Extending the indication as a treatment option for other surface malignancies has been considered. Among these are, for example, endoluminal (urethral) cancer in the bladder and malignant pleural effusion combined with pleural metastasis [17,18]. Combinations of PIPAC with other methods are discussed and tested. Some of these combinations included irradiation [24,25,26], high-intensity ultrasound [27,28,29,30,31], and nanoparticles [32]. Nevertheless, the outcome and patients’ overall survival rate are still unsatisfactory, and the prognosis for patients with disseminated PM remains poor [1,33]. Therefore, there is an urgent need for novel and improved therapeutic concepts. A new concept based on a combination of intraperitoneal hyperthermia beyond 43 °C and peritoneal surface dehydration was recently proposed by Thelen et al. [34] and then further introduced and tested in vivo for its safety and feasibility by Diakun et al. [35,36]. It has been proposed that changing the peritoneal milieu through dehydration and extensive hyperthermia could interfere with PM growth and therefore be an effective tool to reduce PM progression [34,35,37]. Although it has been demonstrated that significant dehydration of the peritoneal cavity in a laparoscopic approach can be achieved, we do not have any data on how cancer cells react to the combined exposure of dehydration, hyperthermia, and chemotherapy in the same setting [36]. Currently, the described effects of peritoneal dehydration under hyperthermic conditions are peritoneal thinning or shrinking and a localized superficial vascular clotting process, which might be highly relevant for targeting neo-vascular structure [36,37]. The effect of this triple therapy (namely dehydration, hyperthermia, and chemotherapy) on cancer cells has never been evaluated. How cancer cells might therefore react under such conditions is unknown but very relevant now (Figure 1). This study will help to evaluate if and to what degree the combined approach will enhance the antitumoral effect. It will further indicate what sequence should be preferably used when applying dehydration, hyperthermia, and chemotherapy all together to achieve the best outcome. Therefore, for the first time, we aim to investigate the potential of dehydration in hyperthermic conditions combined with chemotherapy in various protocols on an HT-29 colon cancer cell line under already established treatment conditions. Although other cell lines would be feasible for the study, the HT-29 colon cancer line is quite resilient and is often used in in vitro peritoneal metastasis models [20,21,22,23]. We further plan to detect and observe changes in intracellular levels of the chemotherapeutic agent under different treatment constellations.

## 2. Results

### 2.1. The Effect of Dehydration under Hyperthermic Conditions Combined with Chemotherapy Compared to Alternatives

**Figure 2:**
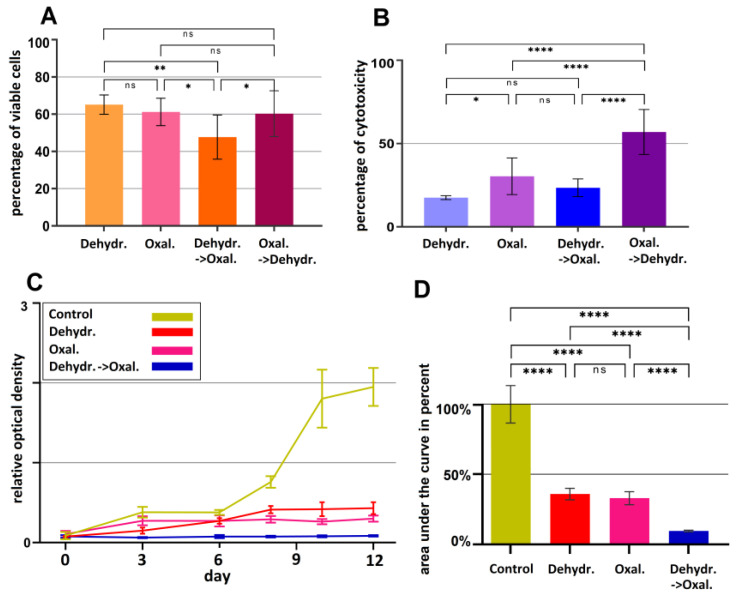
Viability (**A**,**C**,**D**) and cytotoxic levels (**B**) of HT-29 cells in different treatment protocols. (**A**) Viability and cytotoxicity compared to control (**B**) after one cycle of dehydration plus hyperthermia only (1), oxaliplatin only (2), dehydration plus hyperthermia followed by oxaliplatin (3), oxaliplatin followed by dehydration plus hyperthermia (4). (**C**) Development of viability after three cycles of dehydration plus hyperthermia only (red), oxaliplatin only (pink), dehydration plus hyperthermia followed by oxaliplatin (blue). (**D**) The area under the curve of proliferation was calculated, and appropriate statistical analysis was performed for each group (three cycles). Columns include the standard deviation; significance levels are indicated by: * *p* < 0.05, ** *p* < 0.01, **** *p* < 0.0001; ns—not significant.

After the experimental treatments on the HT-29 cell line, their viability significantly decreased when compared to the control (untreated cells). When compared to the control, the viability levels were the following: Cells that were subjected to dehydration plus hyperthermia had a viability level of (65.11 ± 5.15%, *p* < 0.0001) (Figure 2A); when exposed to oxaliplatin alone, viability was at (61.22 ± 7%, *p* < 0.0001); levels for dehydration plus hyperthermia and oxaliplatin were (47.69 ± 12%, *p* < 0.0001); and for the reverse order, namely oxaliplatin and then dehydration plus hyperthermia, viability was at (60.3 ± 12%, *p* < 0.0001).

The cytotoxicity profiles of those groups showed an overall increase in cytotoxicity for all groups. When compared to the control, the cytotoxicity levels were the following: Cells that were subjected to dehydration plus hyperthermia had a cytotoxicity level of (17.54 ± 4%, *p* < 0.0001) (Figure 2B); when exposed to oxaliplatin alone, cytotoxicity was at (30.4 ± 11%, *p* < 0.05); levels for dehydration plus hyperthermia and oxaliplatin were (23.49 ± 5%, *p* < 0.0001). The highest level of cytotoxicity was observed in the group with the reverse order, namely oxaliplatin, followed by dehydration and hyperthermia (56.94 ± 13%, *p* < 0.0001).

### 2.2. The Cumulative Effect of Three-Cycles of Dehydration under Hyperthermic Conditions and Hyperthermia with Chemotherapy Compared to Alternatives

For evaluating the cumulative effect of the proposed therapeutic options on HT-29 cell line proliferation, a total of three treatment cycles were performed and compared to alternatives. Three cycles of dehydration under hyperthermic conditions following oxaliplatin treatment were compared to three cycles of only dehydration and hyperthermia or oxaliplatin (Figure 2C). The integral of the curves over each timepoint was measured and used for analyses because this was a longitudinal observation (Figure 2D).

In comparison with the untreated control, all experimental groups that received any type of treatment showed reduced viability. After three cycles of dehydration under hyperthermic conditions followed by oxaliplatin treatment, cells had significantly lower viability when compared to the control group (4.35 ± 0.5%, *p* < 0.0001). After three cycles of dehydration plus hyperthermia, without chemotherapy, the viability was still significantly reduced at (22.15 ± 4%, *p* < 0.0001). After three cycles of oxaliplatin without any further treatment, the viability was at (15.36 ± 2%, *p* < 0.0001). In comparison, the difference between three cycles of dehydration under hyperthermia with oxaliplatin versus solely oxaliplatin was significant (*p* < 0.0001) (Figure 2D).

### 2.3. The Effect of Dehydration under Hyperthermic Conditions on the Cellular Uptake of Doxorubicin into HT-29 Cell Lines

**Figure 3:**
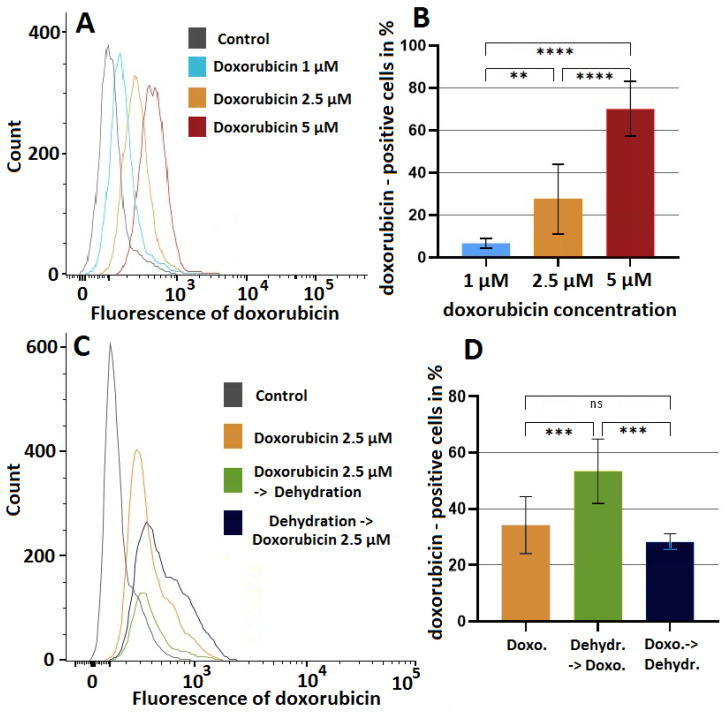
Intracellular doxorubicin uptake from the medium under various protocols. Concentration protocol (**A**,**B**): In A. HT-29 cells, doxorubicin was incubated at concentrations of 1 μM, 2.5 µM, and 5 µM. Intracellular doxorubicin penetration was then analyzed by flow cytometry. Figure B shows the total amount (in percent) of doxorubicin-marked cells for each group. A statistical analysis was performed to compare the number of doxorubicin-marked cells in each group. Treatment protocol (**C**,**D**): flow cytometry (**C**) and histograms (**D**) of HT-29 cells treated with 2.5 µM doxorubicin only, dehydration under hyperthermic conditions followed by doxorubicin, and doxorubicin followed by dehydration under hyperthermic conditions. Untreated cells were used as a control. Results obtained are presented as means ± SD of three independent biological repeats. Significance levels were indicated with: ** *p* <0.01; *** *p* <0.005; **** *p* <0.0001; ns—not significant.

The percentage of doxorubicin-positive cells measured by flow cytometry is dose-dependent and increases with higher dosages of doxorubicin (Figure 3A,B). When looking at the intracellular doxorubicin levels for the different treatment groups, significant differences were detected.

The highest intracellular doxorubicin levels were detected in HT-29 cells, which were first subjected to partial dehydration under hyperthermic conditions and then exposed to doxorubicin. The detected level of doxorubicin-positive cells was 53.35% (vs. both groups, *p* < 0.005). This was significantly higher compared to cells only treated with doxorubicin (34.28%) or cells that were first exposed to doxorubicin and then received dehydration under hyperthermic conditions (28.3%), respectively (Figure 3C,D).

### 2.4. Effect of Dehydration on HT-29 Cells Phenotype Assessed by Phase Contrast Microscopy

**Figure 4:**
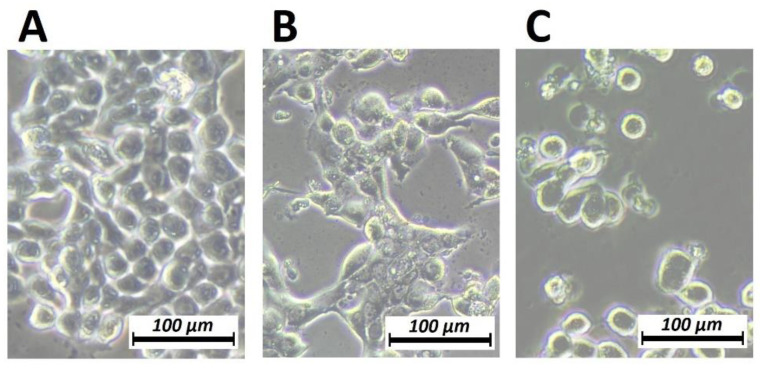
Phase contrast microscopy of HT-29 cells showing the morphology changes and disruption of intercellular connections after one treatment cycle of dehydration in hyperthermic conditions followed by oxaliplatin treatment (**C**). Control cells (**A**) oxaliplatin-treated cells (**B**). 20× magnification level.

After one treatment cycle of partial dehydration under hyperthermic conditions, cells were assessed by phase contrast microscopy to visualize changes in HT-29 cells (Figure 4). In comparison, untreated cells grew as a compacted layer; cells subjected to dehydration and then oxaliplatin treatment were evidently smaller and more disconnected, growing in small groups or as single cells. There were also fewer compared to the control and oxaliplatin groups.

## 3. Discussion

Despite many decades of attempts to improve PM treatment, the results are still not satisfactory. The disease can barely be influenced or slowed down in most patients. Overall median survival rates are about 3–4 months [3,4], with many patients receiving the diagnosis at a late stage where extensive peritoneal metastasis is observed. Progressive morbidity is noted, leading to a wide range of clinical manifestations and complications. The combination of challenges that oncologists and surgeons face does not come close to the therapeutic options at hand. Today, therapeutic options in the management of PM include systemic chemotherapy or locoregional treatments such as intraperitoneal chemotherapy in the form of CRS with HIPAC [8,9] or, for example, PIPAC [4,15]. Different concepts of intraperitoneal chemotherapy are available, and they differ with regard to the way the chemotherapeutic substance is actually delivered and what other physical factors are present at the time of delivery. While HIPEC is characterized by the fact that a hyperthermic solution/lavage is carrying the chemotherapeutic agent, PIPAC is most typically characterized by the fact that the chemotherapeutic agent is delivered via aerosol. 

For the first time now, we have looked at a new approach by adding the parameter dehydration as a major factor into the treatment regime. The cytotoxic effects of chemotherapy on cancer cells are further enhanced by partially dehydrating the cells in a hyperthermic environment. The concept of feasibility of such an approach has already been demonstrated in vivo [35,36,37]. For the first time, we have now investigated the combination of all three therapeutic factors—hyperthermia, dehydration, and chemotherapy—as a possible procedure for PM treatment. Hyperthermia is one therapeutic tool in cancer management, and it has been demonstrated to increase the antitumor effect in many solid and metastatic cancer entities, including PM [38,39]. The novelty of the method presented here goes beyond the factor of dehydration alone. The application of hyperthermia through a gas-based system instead of a liquid-based carrier has a higher likelihood of improvement following laparoscopy. Previous in vivo swine model studies carried out by our research group showed that gas-based intraperitoneal hyperthermia is well tolerated and safe for these animals, and in vitro studies indicate cytotoxic effects on colorectal cancer cell lines, suggesting that this method could be used as a potential tool in PM treatment [35,36]. Based on this, the now presented data is a further indication that combining gas-based hyperthermia with chemotherapy is more cytotoxic on colorectal cancer cells and therefore could also potentially have an additive antitumoral effect in the management of PM than locoregional chemotherapy alone. 

The most pronounced inhibition of colon cancer cell proliferation was detected after three cycles of partial dehydration under hyperthermic conditions, followed by chemotherapy. Proliferation levels decreased around 10-fold compared to control cells and around 3-fold compared to only dehydration or oxaliplatin treatment. The detection of enhanced cellular uptake of chemotherapy after partial dehydration could be one possible explanation for the inhibitory effects seen on cancer cell growth in this study. It remains to be seen if the increased intracellular chemotherapy concentration is the only reason why there is an increased inhibitory effect on the cancer cells. 

This phenomenon can be explained by different mechanisms of cell death. While a decrease in viability measured by the MTS test could indicate apoptosis, elevated cytotoxicity shown by LDH release from permeable cells suggests the release of cell content, which we observe during necrosis or pyroptosis [40]. This information could be of great importance as apoptosis is anti-inflammatory cell death, while necrosis and pyroptosis induce inflammation of surrounding tissues [41,42,43] and, in consequence, cause immune cell influx, which could improve cancer cell eradication or even prevent further metastasis [44,45].

This study demonstrates that the antitumoral effect is not merely reached by the cytostatic drug but can in fact be enhanced by other technical mechanisms such as hyperthermia and dehydration. As of now, how the antitumoral effect of dehydration is played out on a cellular level remains unclear, especially when combined with chemotherapy.

However, in the clinical setting, a potential increased cytotoxic effect on peritoneal metastasis could be reached without increasing the chemotherapeutic concentration. However, as mentioned above, the actual mechanism behind the enhanced antitumoral effect might not be entirely understood at this point. Due to this, an analytical cell study needs to be performed in the future to specifically detect the underlying mechanisms at work, but also an in vivo study needs to be performed for evaluation of aspects such as side effects, safety, and basic applicational features. The results are overall promising; however, further studies need to be conducted to confirm that reproduction can be observed in vivo as well.

This current study is limited as it is in vitro, and some conclusions might not apply in vivo. In addition, the effects of triple therapy need to be assessed in vivo under different circumstances. Further in vivo studies must be conducted that incorporate surgical considerations. These include the ability to perform potential limited surgical resections such as removal of adhesions, intestinal anastomosis, or management of bleeding complications. This aspect will influence which patients could be candidates for triple therapy and which procedures should be avoided simultaneously. This will play a part in the overall assessment of safety and feasibility in an in vivo trial.

Overall, this could significantly increase the efficiency of locoregional chemotherapeutic treatments if the results were similar in an in vivo setting. This proposed concept would be in contrast to HIPEC, a palliative approach similar to PIPAC. Partial surface dehydration and hyperthermia would be performed as an additional supplement to intraperitoneal chemotherapy.

## 4. Materials and Methods

### 4.1. Cell Culture

The human colorectal cancer cell line HT-29 (ATCC^®^ HTB-38™) was obtained from the Institute of Immunology and Experimental Therapy (Wrocław, Poland). HT-29 cells were grown in Dulbecco’s modified Eagle’s medium (DMEM—high glucose, Biowest, Nuaille, France) supplemented with 10% heat-inactivated fetal bovine serum (FBS, Biowest, Nuaille, France), 2 mmol/L glutamine, 100 IU/mL penicillin, and 100 μg/mL streptomycin (all purchased from Sigma-Aldrich; Merck KGaA, Darmstad, Germany) and cultured at 37 °C in a humidified 5% CO_2_/air atmosphere. 

### 4.2. Partial Dehydration under Hyperthermic Condition Combined with Chemotherapy

HT-29 cells were seeded in 24-well plates (TC Plate 24 Well, Standard, F, Sarstedt AG & Co. KG, Nümbrecht, Germany) at a density of 2 × 10^5^ per well and incubated under standard conditions for 72 h before the experiment. The four experimental groups were defined as listed, and an additional untreated group was used as a control. The first group (control) was kept in the full medium at 37 °C. The cells in the second group were subjected to partial dehydration under hyperthermic conditions (30 min at 45 °C in a humidified 5% CO_2_/air atmosphere). In the third group, cells were subjected only to chemotherapy with oxaliplatin (Medoxa, medac GmbH, Wedel, Germany). The cells in the fourth group were subjected to partial dehydration under hyperthermic conditions, and oxaliplatin was added afterward. In the fifth group, cells were subjected to chemotherapy first, followed by partial dehydration under hyperthermic conditions.

The partial cellular dehydration was performed by removing the culture medium from each well and then incubating the cells for 30 min at 45 °C in a humidified 5% CO_2_/air atmosphere. The humidity level was balanced at 85% and kept constant throughout the experiment. In the second group with chemotherapy, only the medium with the chemotherapeutic agent was added, followed by 1h of incubation at 37 °C in a humidified 5% CO_2_/air atmosphere. Afterward, the medium with the chemotherapeutic agent was replaced with pre-warmed DMEM and incubated for 24h until viability assays were performed. Flow cytometry analysis was performed right after the experiment. In those wells that went for further cytometry analysis, doxorubicin (doxorubicin hydrochloride PFS^®^, 2 mg/mL, Pfizer, Sandwich, UK) was added as an additional chemotherapeutic component. Cells dedicated to the viability test received only oxaliplatin at a concentration of 0.24 mg/mL. This corresponds to the implemented dosage for the treatment of peritoneal metastasis of colorectal cancer in HIPEC, whereas cells dedicated for flow cytometry analysis received additional doxorubicin at a concentration of 2.5 µM.

### 4.3. Cumulative Effect of Partial Dehydration under Hyperthermic Condition Combined with Chemotherapy and Proliferation Ability Analysis

In order to measure how partial dehydration in hyperthermic conditions combined with chemotherapy affects the proliferation of HT-29 cells, the following experiments were performed: HT-29 cells were seeded at a density of 1 × 10^3^ in each well and were growing for 72 h. Next, the wells were divided into three experimental groups and one untreated control group. In the control group, cells were kept in a full medium at 37 °C (control). In one group, cells were subjected to only chemotherapy. In another group, cells were subjected to only dehydration under hyperthermic conditions. In the last group, cells were subjected to three cycles of dehydration under hyperthermic conditions, followed by an additional chemotherapeutic treatment at the end of each dehydration cycle. The experimental groups received individual treatments on days 1.5 and 9. The cell viability was constantly measured for a total of 12 days. The integral under the growth curve was measured and statistically compared for statistical analysis.

### 4.4. Analysis the Effects of of Triple-Therapy and Dehydration Using Viability Testing and Cytotoxicity Assay

A viability—MTS test (colorimetric CellTiter 96^®^ AQueous One Solution assay, Promega, Poland) and cytotoxicity—LDH test (Pierce LDH Cytotoxicity Assay Kit, Thermo Scientific, Waltham, Massachusetts, USA) were used to measure the cell effect of triple therapy consisting of partial cellular dehydration in hyperthermic conditions combined with chemotherapy. The MTS test was performed according to the manufacturer’s instructions with certain modifications. The medium was removed from each well and replaced by 0.3 mL of fresh, pre-warmed DMEM. Next, an MTS-based reagent was added to each well, and after 1 h of incubation at 37 °C and 5% CO_2_/air atmosphere, absorbance was detected at 490 nanometers (ղm) using a microplate reader (Tecan, Basel, Switzerland). Cells kept in the medium at 37 °C were used as controls. The percentage of viability was referenced as the control for all groups. The cytotoxicity induced by dehydration and/or oxaliplatin was measured by the release of lactate dehydrogenase (LDH) into the supernatants. A volume of 50 µL of the medium was taken from the wells of each group. The test was performed according to the manufacturer’s protocol. Cytotoxicity levels were calculated as the percentage of LDH released from test samples compared to LDH released by lysis buffer-treated cells and normalized to the spontaneous release from control cells. Absorbance was measured spectrophotometrically on a microplate reader (Tecan, Basel, Switzerland) at 490 nm and 680 nm.

### 4.5. Analysis of Doxorubicin Penetration into HT-29 Cell Line by Flow Cytometry

At first, an assessment of the capability of the dose-dependent detection of doxorubicin in HT-29 cells by flow cytometry was performed. HT-29 cells were exposed to various doxorubicin concentrations. 1 µM, 2.5 µM, and 5 µM doxorubicin were added, and the wells were then incubated for 1h at 37 °C in a 5% CO_2_/air atmosphere. Next, flow cytometry analysis was performed in order to measure the penetration level of doxorubicin into the cells of each of the experimental groups. An untreated group of cells was taken as a control. Right after the experiments, wells were washed twice with phosphate saline buffer (PBS) and trypsin-EDTA (Sigma-Aldrich; Merck KGaA). Thereafter, the detachment of cells was induced. After washing and suspending cells in 0.3 mL of PBS, probes were analyzed by flow cytometry (BD FACS Lyric; Becton Dickinson, Franklin Lakes, NJ, USA). A total of 10000 events of the cell population (gated on Forward Scatter (FSC)—A versus Side Scatter (SSC)—A dot plots) were recorded. The percentage of cells was analyzed by excitation with a 640 nm red laser, and a fluorescence signal was obtained by the detector for R-phycoerythrin (PE) fluorochrome.

The positive signal rate was adjusted based on the autofluorescence of the negative controls. 

### 4.6. Phase Contrast Microscopy

The morphology of control cells and those treated with oxaliplatin and dehydration combined with oxaliplatin was analyzed by phase contrast microscopy. Cells were cultured and treated as stated above, and after 24 h post-exposure to dehydration and chemotherapy, HT-29 cells were visualized by a light microscope with a phase-contrast module using 20-fold lens magnification (Opta Tech, Warsaw, Poland).

### 4.7. Statistical Analysis

All experiments were independently performed three times. All statistical calculations were performed in GraphPad Prism (GraphPad Software, Inc., La Jolla, CA, USA) [39]. A one-way ANOVA test with Tukey’s multiple comparison post hoc test was performed. For the proliferation experiment, the area under the curve (AUC) test was performed. Subsequently, the area from each repetition of the AUC test was taken as a new data table, and an ANOVA with Tukey’s test was performed as stated above. A *p*-value of less than 0.05 was considered statistically significant. The data were presented as the mean standard deviation (SD). * *p* < 0.05; ** *p* < 0.01; *** *p* < 0.005; **** *p* < 0.0001. 

## 5. Conclusions

PM Triple-therapy, consisting of partial dehydration, hyperthermia, and chemotherapy, suppresses cancer cell growth significantly more than either of the factors alone in an in vitro model. This triple therapy needs to be further analyzed for its potential in the treatment of advanced PM. Although the current study is limited to the in vitro characteristics of such a therapy, the initial technical feasibility of such an approach has been confirmed. Beyond the clinical effects, the molecular and biological effects concerning the drug uptake of chemotherapy by cancer cells after dehydration need to be further investigated. Further in-vivo testing needs to be performed to evaluate whether similar encouraging results could be realistic in a clinical setting.

## Data Availability

Data is contained within the article.
